# Supplementary material for: United States County-level COVID-19 Death Rates and Case Fatality Rates Vary by Region and Urban Status
Source: Healthcare (Basel). 2020 Sep 9;8(3):330. doi: 10.3390/healthcare8030330 (PMC7551952; doi:10.3390/healthcare8030330)
Supplement: Supplementary file 1 [file healthcare-08-00330-s001.pdf]

(a)

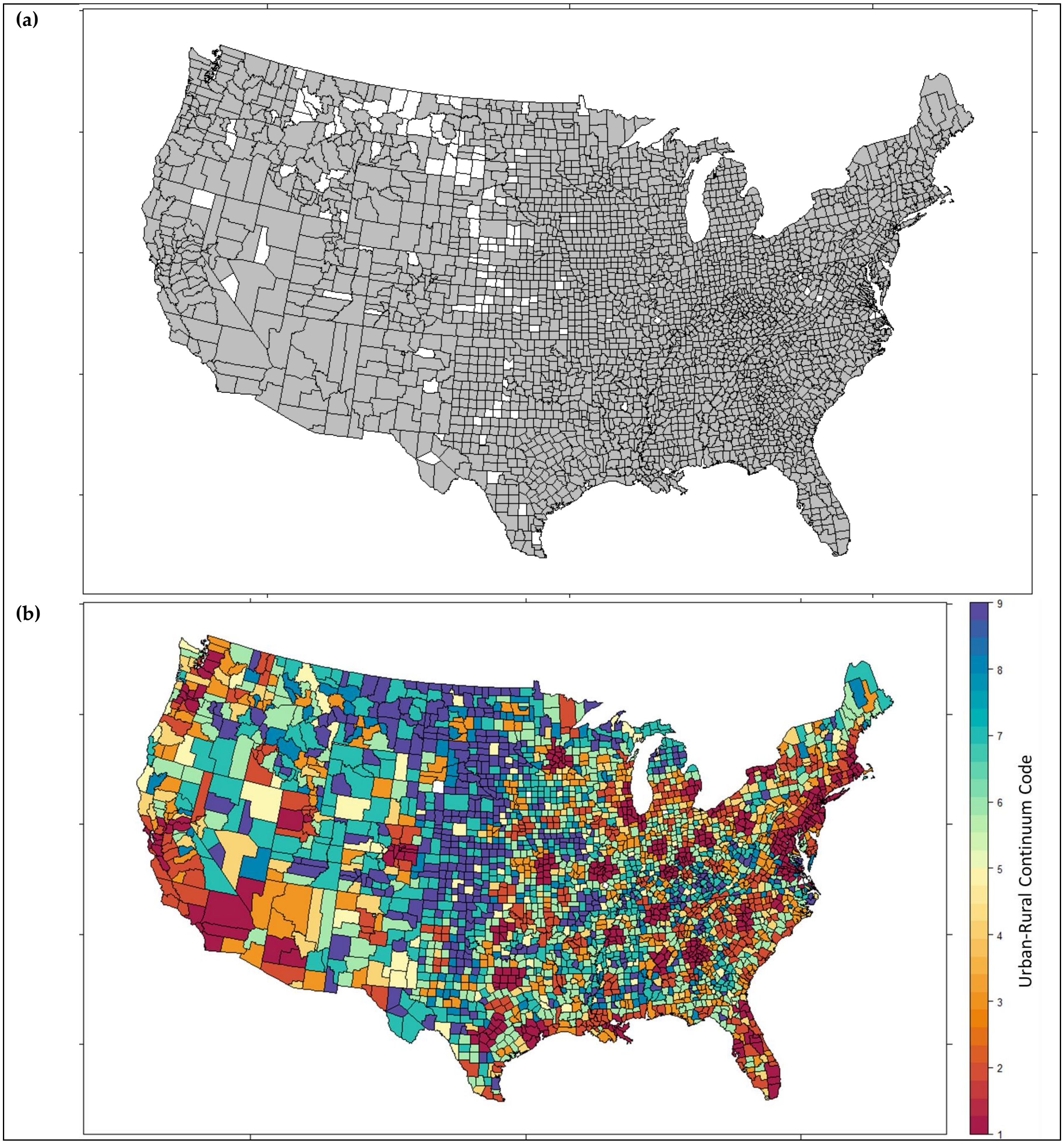

Figure S1. County-level maps. **(a)** COVID-19 case status. Counties in grey had at least one confirmed case by 23 June 2020. Counties in white had not confirmed cases and were not used in the analysis. **(b)** Urban-rural status. Counties were colored based on their 2013 rural-urban continuum code (United States Department of Agriculture Economic Research Service).

Table S1. Null and full model significance and fit statistics. Significance values and fit statistics for both null (variable(s) of interest only) and full models (variable(s) of interest and confounding factors).

	**Null Model**				**Full Model**			
	**F-Value**	***p*-Value**	**Pearson Chi-Squared/DF**	**AICc Score**	**F-Value**	***p*-Value**	**Pearson Chi-Squared/DF**	**AICc Score**
Deaths per 100,000								
**Rural-Urban**	19.72	<0.001	1.19	19886.36	10.56	<0.001	1.20	19532.88
**Region**	50.93	<0.001	0.98	19818.26	19.37	<0.001	1.21	19530.05
**Interaction**	-	-	-	-	4.45	<0.001	1.29	19408.39
Deaths per cases								
**Rural-Urban**	12.61	<0.001	1.31	-7677.06	7.32	<0.001	1.30	-7692.80
**Region**	19.20	<0.001	1.47	-7559.42	17.35	<0.001	1.32	-7714.03
**Interaction**	-	-	-	-	3.14	<0.001	1.25	-7780.82

Table S2. Population mortality rate estimates for interaction model. Model-corrected mean mortality rate per 100,000 for the interaction between US region and Rural-Urban Continuum Code. Confidence Intervals are 95%.

Rural-Urban Code	US Region	Mean	Lower CI	Upper CI
1	Midwest	27.0286	19.003	38.4437
2	Midwest	15.8208	10.6489	23.5045
3	Midwest	19.8212	14.1444	27.7765
4	Midwest	21.052	13.9772	31.7077
5	Midwest	13.5222	7.4193	24.6452
6	Midwest	18.1381	14.0858	23.3561
7	Midwest	10.7542	8.1033	14.2721
8	Midwest	13.5124	8.874	20.5751
9	Midwest	2.5257	1.8572	3.4349
1	Northeast	54.1714	33.9185	86.5175
2	Northeast	44.4105	26.3462	74.8606
3	Northeast	27.7557	14.4771	53.2136
4	Northeast	17.7833	9.3515	33.8177
5	Northeast	3.939	0.3149	49.2679
6	Northeast	16.0482	8.6996	29.6042
7	Northeast	2.862	1.2247	6.688
8	Northeast	2.9336	0.6537	13.1659
9	Northeast	0.000015	1.08×10^{-153}	2.22×10^{143}
1	Southeast	12.3538	9.2226	16.5481
2	Southeast	8.8594	6.7271	11.6676
3	Southeast	12.6128	9.3781	16.9633
4	Southeast	8.4136	5.3947	13.1221
5	Southeast	11.9758	5.6148	25.5431
6	Southeast	13.45	10.4369	17.3331
7	Southeast	12.4522	8.8415	17.5375
8	Southeast	8.0411	5.4204	11.9289
9	Southeast	9.9245	6.5864	14.9542
1	Southwest	4.9543	2.8597	8.583
2	Southwest	8.2249	4.6702	14.4853
3	Southwest	11.9765	6.3992	22.4147
4	Southwest	14.2088	6.7676	29.8317
5	Southwest	15.5039	6.0647	39.6348
6	Southwest	8.6809	5.8062	12.9787
7	Southwest	7.2489	4.4667	11.7641
8	Southwest	4.6357	2.01	10.6912
9	Southwest	4.4569	2.4267	8.1854
1	West	10.0353	5.4573	18.4535
2	West	7.688	4.4391	13.3146
3	West	7.0635	4.0683	12.2638
4	West	3.3839	1.6676	6.8669
5	West	4.6738	2.1044	10.3803
6	West	7.8265	4.388	13.9594
7	West	11.6106	7.7199	17.4621
8	West	1.8717	0.6598	5.31
9	West	1.7126	0.9482	3.093

Table S3. Case fatality rate estimates for interaction model. Model-corrected mean case fatality rate percentage (CFR%) for the interaction between US region and Rural-Urban Continuum Code. Confidence Intervals are 95%.

Rural-Urban Code	US Region	CFR %	Lower CI	Upper CI
1	Midwest	5.7	5.0	6.5
2	Midwest	4.8	4.1	5.7
3	Midwest	4.5	3.8	5.2
4	Midwest	4.3	3.6	5.1
5	Midwest	4.6	3.5	6.1
6	Midwest	5.9	5.3	6.7
7	Midwest	5.5	4.7	6.5
8	Midwest	9.8	8.1	11.8
9	Midwest	8.0	6.3	10.2
1	Northeast	7.4	6.3	8.5
2	Northeast	6.4	5.4	7.7
3	Northeast	5.6	4.4	7.1
4	Northeast	4.0	3.1	5.2
5	Northeast	3.0	1.0	8.1
6	Northeast	4.6	3.5	6.0
7	Northeast	4.5	3.0	6.8
8	Northeast	4.7	2.0	10.7
9	Northeast	9.4	4.9	17.2
1	Southeast	4.3	3.8	4.8
2	Southeast	3.8	3.4	4.3
3	Southeast	4.2	3.7	4.8
4	Southeast	3.4	2.8	4.1
5	Southeast	4.7	3.4	6.3
6	Southeast	4.2	3.8	4.7
7	Southeast	4.2	3.6	4.9
8	Southeast	4.3	3.6	5.3
9	Southeast	5.2	4.2	6.4
1	Southwest	4.7	3.6	6.0
2	Southwest	5.7	4.5	7.3
3	Southwest	5.0	3.7	6.7
4	Southwest	5.2	3.8	7.1
5	Southwest	6.2	4.3	8.9
6	Southwest	6.0	5.0	7.3
7	Southwest	7.2	5.5	9.4
8	Southwest	9.3	6.0	14.0
9	Southwest	22.8	17.3	29.4
1	West	5.4	4.2	7.0
2	West	4.7	3.6	6.0
3	West	4.9	3.8	6.2
4	West	3.7	2.4	5.7
5	West	4.3	2.5	7.1
6	West	5.1	3.6	7.2
7	West	6.0	4.7	7.7
8	West	3.9	1.4	10.1
9	West	7.0	3.8	12.4
